# Nuclear Weapons 60 Years On: Still a Global Public Health Threat

**DOI:** 10.1371/journal.pmed.0020301

**Published:** 2005-08-23

**Authors:** Rhona MacDonald

## Abstract

This year marks the 60-year anniversary of the bombings of Hiroshima and Nagasaki. Has the world learned anything about the threat of nuclear weapons? What role can the health community play in reducing it?

The United States carried out the world's first nuclear test, codenamed “Trinity,” on 16 July 1945 in the desert of New Mexico. Just three weeks later, on 6 August, the US exploded a uranium device called “Little Boy” 2,000 feet above the Japanese city of Hiroshima, killing around 150,000 people. Three days later, the US deployed a second nuclear bomb, a plutonium device called “Fat Man,” that exploded above the Japanese city of Nagasaki, resulting in at least 74,000 deaths. These two terrible acts heralded the start of the nuclear age, which reached its peak during the Cold War.

Sixteen years after the fall of the Berlin wall, symbolising the end of the Cold War, it is easy to forget the terror that gripped the world over the threat of a nuclear war that could destroy the planet. But the state of the world at the 60-year anniversary of the bombing of Hiroshima should be enough to shake us from our complacency, especially considering the number of countries that have nuclear weapons and haven't signed or ratified the relevant treaties, and the outcome of the latest review of the Non-Proliferation Treaty (NPT) earlier this year (see below).

## Who Has Nuclear Weapons?

Russia, the US, the United Kingdom, France, and China are the only “declared” nuclear states; that is, they have declared that they have nuclear weapons in the NPT. But there are also “undeclared” states. Table 1 shows the countries that have nuclear weapons and how many they have. Box 1 shows the current status of these countries and in what situation they have indicated they would be prepared to use their nuclear weapons.

**Box 1. Current Status of Nuclear States**

**UK**

- One of the four Trident submarines is on patrol at all times.
- The missiles are not targeted (that is they are not aimed at a specific target) and are normally at several days notice to fire.
- Accepts a first-use policy. That means that, in certain circumstances, it is prepared to use nuclear weapons first. Under the Conservative Government there was a policy of using nuclear weapons to protect Britain's ‘vital interests’. That policy has never been changed. In the build-up to the Iraq war in 2003, Defence Secretary Geoff Hoon said that the UK would use nuclear weapons if its troops were attacked with chemical or biological weapons.
- The UK opposes a time-bound framework for disarmament. ‘Time-bound’ means putting deadlines on when agreed things have to be achieved. It also voted against multilateral negotiations proposed at the UN General Assembly.

**France**

- One submarine is on patrol at all times.
- Although policy is vague, France has never supported ‘no first-use’ and has said that it would use nuclear weapons to defend its ‘vital interests’.
- Opposes a time-bound framework and a multilaterally negotiated nuclear disarmament convention.

**US**

- At least ten submarines are on patrol at all times.
- The Nuclear Posture Review of 2002 gave examples of when the US would use nuclear weapons first.
- Opposes multilateral negotiations
- Opposes including nuclear weapons on the International Court of Justice list of prohibited weapons.

**Russia**

- Estimated that at least two submarines on patrol at all times.
- Russia has a ‘no first-use’ policy.
- Supports multilateral negotiations.

**China**

- Has a ‘no first-use’ policy and has also said that it would not use nuclear weapons against a non-nuclear weapon state.
- Supports a time-bound framework and has called for a convention banning nuclear weapons.

**India**

- Has a ‘no first-use’ policy and has said that it would not use nuclear weapons against a non-nuclear weapon state.
- Supports a nuclear weapons convention and sponsors a resolution at the UN on de-alerting nuclear weapons.

**Pakistan**

- Has said that it would use nuclear weapons first in a conflict.

**Israel**

- Will not confirm or deny having nuclear weapons.
- Says that it will not be the first to introduce nuclear weapons into the Middle East but will not explain exactly what it means by that.
- Opposes a time-bound framework.

Box text quoted from [5].

**Table 1:**
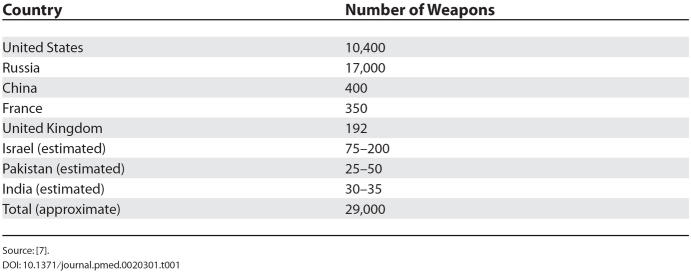
Countries That Have Nuclear Weapons

Source: [7].

## The Crucial Treaties

### The NPT

The main objective of the NPT is to stop the spread, or “proliferation,” of nuclear weapons. The declared nuclear states had to agree not to pass on to other countries any nuclear weapons technology and, under Article VI, they also have to “pursue negotiations in good faith on effective measures relating to the cessation of the nuclear arms race at an early date and to nuclear disarmament….” Non-nuclear-weapon states had to promise not to make any attempt to acquire nuclear weapons. If they complied, in return they could get help to develop a nuclear power programme. Box 2 shows who has not signed the NPT.

**Box 2. The NPT**

- The treaty opened for signature in 1968.
- It entered into force in 1970.
- A total of 188 countries have signed.
- India, Pakistan, Israel, Cook Islands, and Niue have not signed.
- The Democratic People's Republic of Korea (North Korea) announced its withdrawal from the NPT in January 2003.

Source: [2].

The US recently granted India access to its civilian nuclear knowledge in exchange for a “global partnership.” India is not a signatory of the NPT, so is not bound by its provisions, and it has always been American foreign policy, upheld by law, that only countries that are NPT members should share any benefits of American civilian nuclear expertise, so this is a worrying development [1].

### Comprehensive Test Ban Treaty

After years of negotiations, the Comprehensive Test Ban Treaty was overwhelmingly endorsed in 1996 at the United Nations in New York. To date, it has been signed by 167 countries and ratified by 99. The Comprehensive Test Ban Treaty outlaws nuclear testing of any kind and must be signed and ratified by the 44 countries identified as having nuclear power plants or research reactors. Ten of those countries have signed but not ratified: Algeria, China, Colombia, Democratic Republic of Congo, Egypt, Indonesia, Iran, Israel, the US, and Viet Nam. Three have not signed it at all: India, Pakistan, and North Korea [2].

## The Seventh Review of the NPT

There was some optimism after the sixth NPT review in May 2000, with the media reporting that a nuclear-free world was in sight. There were 13 points that all participant countries agreed to adhere to in time for the next convention (see Box 3). When the UK's Minister of Defence, Geoff Hoon, said a few months later, “The NPT agreement is an aspiration; it is not likely to produce results in the short term” [3], it was a sign of things to come.

**Box 3. The 2000 NPT Review Conference Final Document**

A ‘Programme of Action’ (often referred to as the ‘13 practical steps towards global nuclear disarmament’) became part of the Final Document. They are summarised as:

(1) Progress needs to be made on entry-into-force of the Comprehensive Test Ban Treaty (CTBT).

(2) The moratorium on nuclear weapon test explosions must be maintained.

(3) The Conference on Disarmament (CD) must move forward in establishing a Fissile Material Cut-Off Treaty (FMCT).

(4) A subsidiary body with a mandate to deal with nuclear disarmament is needed.

(5) The principle of irreversibility on arms control and reduction agreements must be applied to nuclear disarmament measures.

(6) Progress on nuclear disarmament (implementation of Article VI) is required.

(7) Implementation of arms reduction agreements and pursuit of binding agreements on further irreversible reductions must be instituted.

(8) Greater emphasis must be attached to the implementation of the Trilateral Initiative and greater support must be forthcoming for the International Atomic Energy Agency (IAEA).

(9) Confidence building measures and progressive steps to lower the nuclear threshold must be offered. [These include increased effort by the NWS to reduce their nuclear arsenals unilaterally; increased transparency by the NWS about their nuclear weapons capability; further reductions of non-strategic nuclear weapons; a reduction in the operational status of nuclear weapons (de-alerting); a diminished role for nuclear weapons in security policies (doctrines); and the engagement of all NWS in facilitating the elimination of nuclear weapons.]

(10) Further fissile material stocks must be put under IAEA Safeguards.

(11) The ultimate objective of complete nuclear disarmament must be reaffirmed.

(12) The formal reporting back by States Parties between Review Conferences— the accountability principle—must be instituted.

(13) Enhanced verification measures must be agreed and implemented.

Box text quoted from [6].

This May, after a month of arguments, the seventh NPT review ended in failure. According to Gunnar Westberg and John Loretz, Co-President and Program Director, respectively, of International Physicians for the Prevention of Nuclear War (IPPNW): “At the end of the day, the review collapsed over one issue: the refusal of the United States to build on the foundations for disarmament that were laid in 1995 and 2000, or even to acknowledge that those foundations exist” [4].

They continue: “The Bush administration may attempt to spin the meaning of the failed NPT review to suit its distaste for multilateral negotiations and for the UN as an institution. This would be akin to a teenager breaking the lawnmower and then telling his parents that he can't cut the lawn because the lawnmower doesn't work. One cannot deliberately break a consensus-based decision making process and then claim that multilateralism does not work” [4].

Michael Christ, Executive Director of IPPNW, told *PLoS Medicine*: “The failure of the 2005 NPT review exposed the underlying realities that stand as obstacles to achieving a world free of nuclear weapons: firstly, a stubborn refusal by the nuclear weapon states, particularly the US, to comply with their disarmament commitments and, conversely, an insistence that nuclear weapons are indispensable to their security and to the pursuit of their global interests; secondly, increasing levels of frustration and impatience among non-nuclear-weapons states, the overwhelming majority of which want nuclear disarmament; and thirdly, the increasingly unavoidable and dangerous contradiction between guaranteeing access to ‘peaceful’ uses of nuclear energy while at the same time ensuring that such uses do not become a platform for weapons development.”

## Where Does That Leave the World in 2005?

In the current climate of increased global terrorism, the aftermath of the war in Iraq, and the uncertain situation in Iran and North Korea regarding nuclear weapons, where does the failure of the 2005 NPT review leave us?

Douglas Holdstock from the campaigning organisation Medact, said: “North Korea may have five to ten usable weapons. It is very unlikely to use them until directly attacked by the US. Iran will not have usable nuclear weapons for about five years.”

“The greatest risks of use,” he said, “are probably (1) India and Pakistan over Kashmir; (2) Israel against any nearby Islamic state, particularly if attacked by chemical or biological weapons; (3) India and China over border disputes; and (4) China and the US, for example, over Taiwan. Any of these could kill millions and cause widespread fallout, but probably not ‘nuclear winter’. This would only follow a US–Russia exchange, which is a low risk at present—but will remain as long as nuclear weapons exist.”

Michael Christ explained that about 5,000 US and Russian nuclear weapons are still on 24-hour hair trigger alert, ready to be launched at a moment's notice: “These are fallible machines being operated by fallible human beings—and we have had a number of frighteningly close calls with accidental nuclear war. Our [IPPNW's] calculations indicate that nearly 7 million Americans would die immediately from an accidental launch of weapons from a single Russian submarine. Furthermore, some of the nuclear powers, led by the US, are planning for a new generation of ‘useable’ battlefield nuclear weapons—‘bunker busters’ and ‘mini-nukes.’”

Gunnar Westberg thinks that having nuclear weapons is contagious: “If Russia and the USA say they need nuclear weapons for their security, of course smaller countries will feel the same, with stronger reasons. If the nuclear-weapons states do not abolish their arsenals, proliferation to many more countries cannot be stopped.”

The Campaign for Nuclear Disarmament argues that counter-proliferation methods are replacing the concept of non-proliferation. Ruth Tanner, Press Officer for the Campaign for Nuclear Disarmament UK, said: “The concept of non-proliferation, as enshrined in the NPT, is under threat from the drive by the US and UK towards a policy of counter-proliferation, rather than non-proliferation. Counter-proliferation policies also further undermine the multilateral non-proliferation regime through its possible substitution—as in the case of Iraq—by pre-emptive disarmament wars, carried out by a tiny minority of the international community.”

“Missile defence,” she said, “is clearly part of the counter-proliferation approach, for it enables first strike without fear of retaliation.”

## Impacts on Health

“There are the enormous impacts on health and environment, documented in numerous studies, resulting from the development, manufacture, testing, stockpiling, maintenance, transport, dismantling, storage, and disposal of nuclear weapons,” said Michael Christ. “Every one of these steps poses direct risks to the health of the personnel involved and the general population. We [IPPNW] estimated 430,000 deaths worldwide from fatal cancer as a consequence of US atmospheric nuclear testing, from 1945 to 1963. Nuclear programs worldwide have left behind a toxic legacy that will affect human health and the environment for thousands of years. In the US alone, this folly cost taxpayers $5.5 trillion between 1940 and 1996. And spending is on the rise.”

He explained what IPPNW is doing to publicise the threat of nuclear weapons: “We are emphasizing the medical and moral imperative of nuclear disarmament. We must stigmatize nuclear weapons not on the basis of who owns them but for what they are and what they can do. These are not weapons at all—they are instruments of indiscriminate mass murder. They are Nazi crematoria mounted on missiles.”

The world's major health problems are all related, and are ultimately affected by how much money is spent on weapons, according to Douglas Holdstock: “Poverty, under-development, disease, [and] war, which [are] fuelled by the arms trade, climate change, and other environmental threats, such as over-population, are all inter-linked.” And reducing nuclear and other arms spending will free resources for better causes, he said.

## What Can International Health-Care Workers Do?

Michael Christ reminds us what is at stake: “We are moving inexorably towards a major nuclear disaster of some form, and the medical dangers are just too profound to ignore for those concerned about and responsible for public health.”

He continued: “The heart of the problem is a lack of political will to rid the world of the only weapons that could extinguish most life on earth in a matter of hours. Creating that political will is our focus for the future.”

Douglas Holdstock said, “[Nobel Peace Laureate] Sir Joseph Rotblat says that to prevent nuclear war we must prevent all war, as the knowledge of how to make nuclear weapons will indeed always be with us. Rotblat ended his Nobel Prize acceptance speech by saying, ‘remember your humanity.’”

“At this difficult and dangerous time it is vital that we work for peace in the world,” said Ruth Tanner. “Nuclear weapons are a threat to the planet and its people and the rogue states that insist on maintaining their destructive arsenal are a minority in a world that wants to be free of nuclear weapons.”

“The NPT is still valid,” said Gunnar Westberg. “A strong international movement for a nuclear weapons convention, prohibiting nuclear weapons, is needed, and may be developing just now. It may work along the pattern of the Campaign to Ban Landmines.”

He continued: “We [physicians] are used to talking to people about questions of life and death. So we must tell the general public that nuclear weapons are the greatest threat to the survival of mankind, and the only intervention that will work is the complete abolition of all nuclear weapons. Now is the time to do this, in this period of low tension between the big powers, and before nuclear weapons proliferate to many more countries.”

“Nuclear weapons and mankind,” he added, “can in the long run not coexist. Either will be abolished. We have a choice.”

## Conclusion

The world is in turmoil: terrorism, or at least the fear of terrorism, seems to have a stranglehold; world governments and the United Nations have an arbitrary way of dealing with “rogue states” (notice, for example, the differences in their treatment of Iraq, the Democratic Republic of Congo, Zimbabwe, and Myanmar); and international treaties can be broken on a whim, such as the recent deal in which the US agreed to share its civilian nuclear knowledge with India. Now is not the time to be gambling with the world's future and that of the human race by holding on to weapons that could destroy the planet thousands of times over. The countries that continue to have such weapons are potential destroyers, not the guardians of democracy, or the defenders of peace, or whatever they choose to call themselves. Democracy should be better than this.

**Useful Links**

**International Physicians for the Prevention of Nuclear War:** www.ippnw.org

**Medact:** www.medact.org

**Campaign for Nuclear Disarmament:** www.cnduk.org

**WMD Awareness Programme:** www.comeclean.org.uk

**British American Security Information Council:** www.basicint.org

## Abbreviations

IPPNW: International Physicians for the Prevention of Nuclear War
NPT: Non-Proliferation Treaty
NWS: nuclear weapons states
